# Element substitution of kesterite Cu_2_ZnSnS_4_ for efficient counter electrode of dye-sensitized solar cells

**DOI:** 10.1038/s41598-018-26770-1

**Published:** 2018-06-07

**Authors:** Shuang Lu, Huanying Yang, Fei Li, Yinglin Wang, Shixin Chen, Guochun Yang, Yichun Liu, Xintong Zhang

**Affiliations:** Center for Advanced Optoelectronic Functional Materials Research, and Key Lab of UV-Emitting Materials and Technology of Ministry of Education, Northeast Normal University, 5268 Renmin Street, Changchun, 130024 China

## Abstract

Development of cost-effective counter electrode (CE) materials is a key issue for practical applications of photoelectrochemical solar energy conversion. Kesterite Cu_2_ZnSnS_4_ (CZTS) has been recognized as a potential CE material, but its electrocatalytic activity is still insufficient for the recovery of I^−^/I_3_^−^ electrolyte in dye-sensitized solar cells (DSSCs). Herein, we attempt to enhance the electrocatalytic activity of kesterite CZTS through element substitution of Zn^2+^ by Co^2+^ and Ni^2+^ cations, considering their high catalytic activity, as well as their similar atomic radius and electron configuration with Zn^2+^. The Cu_2_CoSnS_4_ (CCTS) and Cu_2_NiSnS_4_ (CNTS) CEs exhibit smaller charge-transfer resistance and reasonable power conversion efficiency (PCE) (CCTS, 8.3%; CNTS, 8.2%), comparable to that of Pt (8.3%). In contrast, the CZTS-based DSSCs only generate a PCE of 7.9%. Density functional theory calculation indicate that the enhanced catalytic performance is associated to the adsorption and desorption energy of iodine atom on the Co^2+^ and Ni^2+^. In addition, the stability of CCTS and CNTS CEs toward electrolyte is also significantly improved as evidenced by X-ray photoelectron spectroscopy and electrochemical impedance spectroscopy characterizations. These results thus suggest the effectiveness of the element substitution strategy for developing high-performance CE from the developed materials, particularly for multicomponent compounds.

## Introduction

High-efficiency, good-stability and low-cost counter electrodes (CEs) are essential for photoelectrochemical solar energy conversion. As a key component of the photoelectrochemical solar cells, the CEs need to possess good conductivity and high catalytic activity for the efficient recovery of redox. Up to now, Pt is the most widely applied CE active materials for dye-sensitized solar cells (DSSCs)^1,2^. However, the high cost of Pt-based materials limits their further development. Numerous candidates are exploited to replace the expensive Pt, such as metals and alloys^3^, carbon materials^4,5^, conductive polymer^6,7^, transition metal compounds^8–10^ and composites^11,12^. Among them, the transition metal compounds (TMCs) attract much attention because of their Pt-like catalytic activity^13–19^. Various binary TMC CEs are wildly investigated, however the study of multicomponent TMC CEs is still limited despite they have many advantages, such as material diversity and multiple activity sites^19^.

Recently, kesterite Cu_2_ZnSnS_4_ (CZTS), a quaternary transition metal sulfide, is considered to be a promising photo- and electro-catalyst due to its tunable band gap (1.0–1.5 eV), high abundance and nontoxicity^20–29^. After optimizing composition and morphology of CZTS CE, the efficiency of DSSCs was reported in the range from ~4% to 9%. But, the catalytic activity of CZTS is still limited, due to its fully-filled d orbitals of metallic active sites (Zn^2+^ and Sn^4+^)^30^. Thus, it is reasonable to suppose that the substitution of Zn^2+^ or Sn^4+^ by more active metal ions would enhance the activity of CZTS CE. Co^2+^ and Ni^2+^ are high-activity catalytic sites in various photo- and electro-catalysts^31–38^. Series of highly efficient CE materials based on Co^2+^ and Ni^2+^ have been exploited, including carbides^39^, nitrides^40^, chalcogenides^41,42^ and oxides^43^. Furthermore, these two divalent metal ions present similar atomic radius and electron configuration with Zn^2+^, thus substituting Zn^2+^ by Co^2+^ or Ni^2+^ may improve the catalytic activity of CZTS.

Herein, we investigate the effect of element substitution on improving the electrocatalytic activity of kesterite CZTS CEs. We prepare kesterite Cu_2_XSnS_4_ (X = Zn, Co, Ni) CEs by simple spin-coating method. Electrochemical impedance spectroscopy (EIS) and X-ray photoelectron spectroscopy (XPS) tests indicate that the Cu_2_CoSnS_4_ (CCTS) and Cu_2_NiSnS_4_ (CNTS) CEs possess decreased change-transfer resistance and improved stability toward iodide electrolyte. CCTS- and CNTS-based DSSCs exhibit enhanced efficiency (8.3% and 8.2%) compared with that of CZTS (7.9%), which is comparable with traditional Pt (8.3%). In addition, the highly-effective catalytic activity is related to the adsorption and desorption energy of iodine (I) atom calculated by the density functional theory^44–47^.

## Results and Discussion

### Structure and morphology characterizations

**Figure 1:**
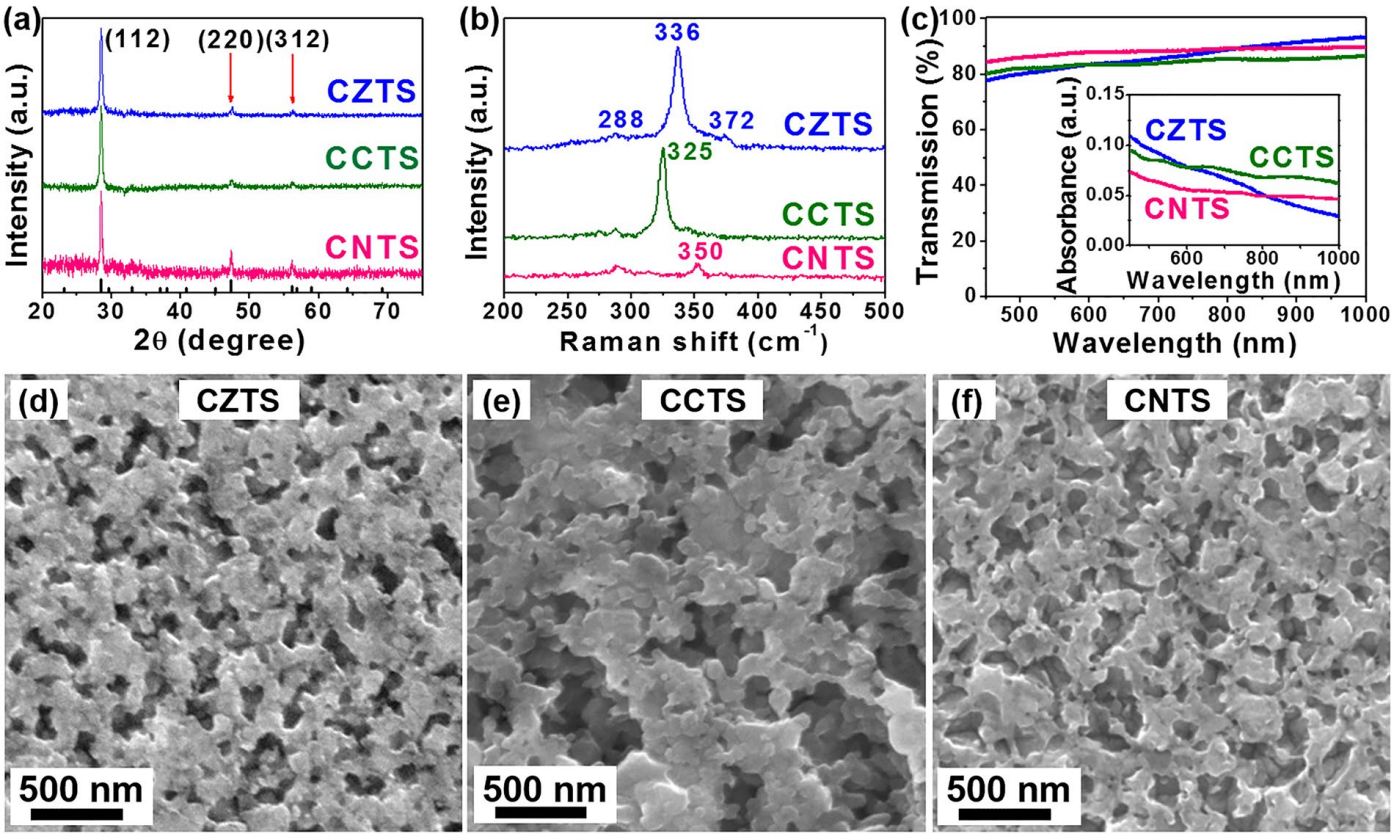
(**a**–**c**) XRD patterns, Raman and UV-Vis spectra and (**d**–**f**) top-view SEM images of CXTS films prepared by H_2_O/ethanol precursor solutions. The films of XRD and Raman measurements were prepared on quartz.

We prepared porous CXTS films by spin-coating precursor solutions based on water and ethanol mixed solvent and annealing them in N_2_ atmosphere at 540 °C for 15 minutes^48^. To avoid the signals interference of FTO (SnO_2_: F) to CXTS films, we recorded X-ray diffraction (XRD) patterns and Raman spectra through CXTS films on quartz prepared by the same method. The diffraction peaks at 28.53°, 47.33°, and 56.18° were indexed to (112), (220), and (312) planes respectively, which were in good agreement with those of previously reported kesterite CZTS^48–50^ (Fig. 1(a)). This measurement indicated that the element substitution did not change the crystal structure of CZTS. Furthermore, three peaks at 288, 336 and 372 cm^−1^ were observed in the Raman spectra (Fig. 1(b)) of CZTS, which were indexed to CZTS materials. The CCTS and CNTS spectra showed peaks at 288, 325 and 350 cm^−1^, which were observed in the CCTS and CNTS materials of previous literatures^51–54^. In addition, we used Energy-dispersive X-ray spectroscopy (EDX) to analyze the composition of CXTS films (Fig. S1(a–c) in the Supplementary Information). The elemental composition ratio was 1.8:1:1.3:4.7, 1.5:1:1.3:4.4 and 1.5:1:1.1:4, respectively. These results indicated that the CXTS CEs was successfully synthesized. In addition, CXTS CEs showed above 75% transmittance in the range of visible wavelengths as shown in the UV-Vis spectra of Fig. 1(c).

Figure 1(d–f) exhibited the top-view scanning electron microscope (SEM) images of the CXTS films. It was obvious that the CXTS films showed a porous structure, which was beneficial to the high catalytic activity because of the high specific surface area^55^. The Atomic force microscope (AFM) measurements also showed similar morphology (see Fig. S1(d–f) in the Supplementary Information). We performed step profiler test to accurately measure the thickness of CXTS films. The thickness of CZTS, CCTS and CNTS CEs were calculated to be 189 ± 13 nm, 125 ± 3 nm and 148 ± 27 nm, respectively, from nine measure points. The thickness of CXTS films was carefully optimized by spin-coating 1 layer of precursor solution (see Fig. S2 in the Supplementary Information) providing enough active sites and reduced bulk resistance.

### Electrochemical characterization

**Figure 2:**
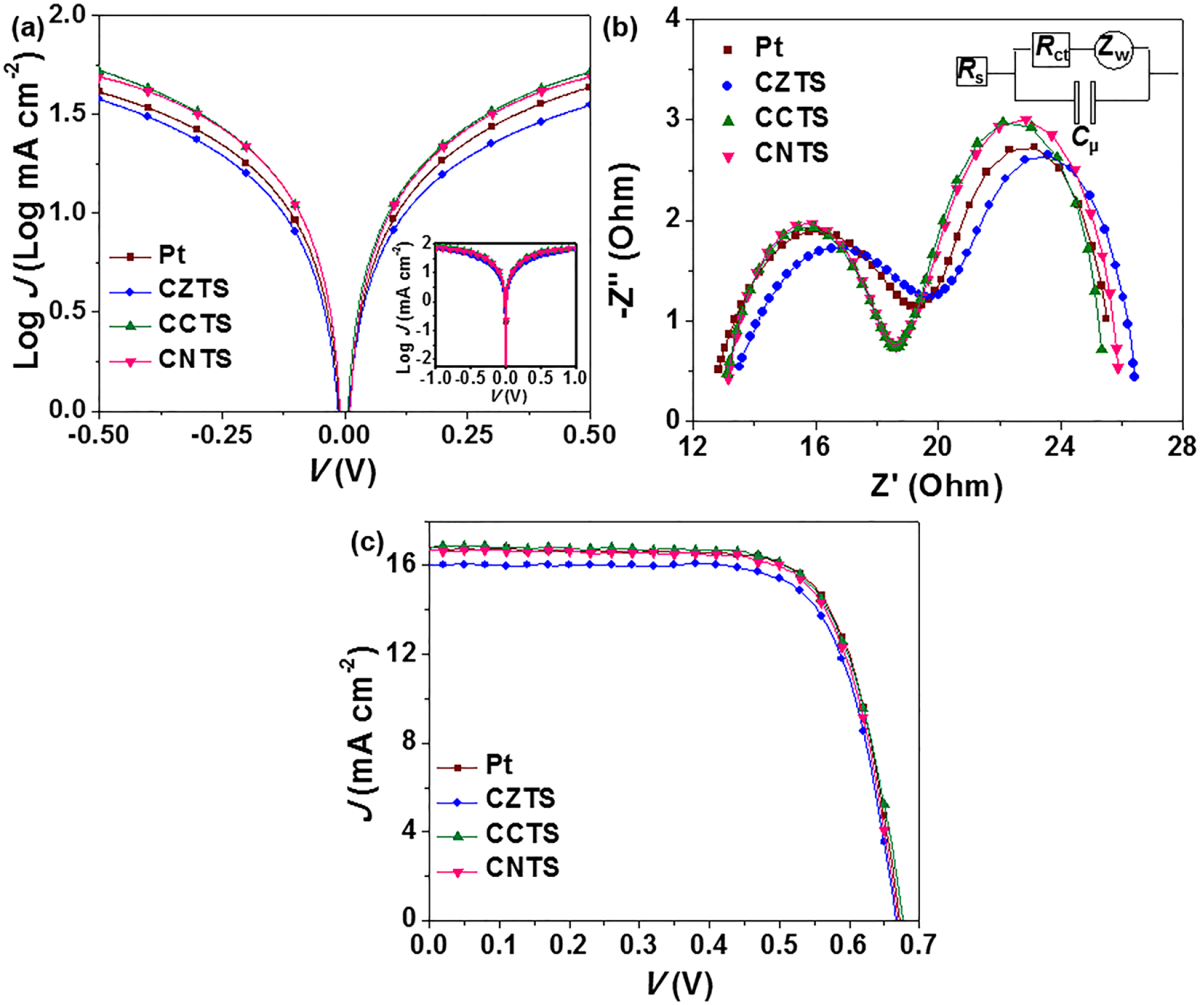
(**a**) Tafel curves and (**b**) EIS plots of Pt and CXTS CEs. Both Tafel and EIS experiments were performed with the symmetrical dummy cells with two identical electrodes (CE//iodide electrolyte//CE). Inset in a and b show the original Tafel curves and the equivalent circuit model of the symmetrical cells for fitting EIS results. (**c**) *J*–*V* curves of DSSCs based on Pt and CXTS CEs, measured under AM 1.5 G solar simulator illumination (100 mW cm^−2^).

To investigate the electrocatalytic activity of kesterite CXTS CEs for reducing iodide electrolyte, we performed Tafel polarization measurements with the symmetrical structure (CE//electrolyte//CE). The exchange current density (*J*_0_) of CEs could be acquired from the intercept of a tangent to Tafel polarization curves (Tafel), the variation of which could be inverse with the charge-transfer resistance (*R*_ct_) values fitted from EIS through eq. 1:

1 $${J}_{0}=\frac{RT\,}{nF{R}_{{\rm{ct}}}}$$

where *R* is the gas constant, *T* is the temperature, *F* is Faraday’s constant and *n* is the electron number involved in the electrochemical reduction of triiodides at the electrode^56^. As shown in Fig. 2(a), the anodic and cathodic branches of CCTS- and CNTS-Tafel curves exhibited larger slopes than those of CZTS, revealing a higher *J*_0_ and more efficient catalytic activity of CCTS and CNTS CEs for reducing triiodides. In addition, we also prepared Cu_2_MnSnS_4_ and Cu_2_FeSnS_4_ CEs by similar method. However, their performance was poor because of the bad activity and unstable chemical property of these two films.

The EIS test was used to further evaluate the catalytic activity of CXTS CEs. The left arcs of EIS spectra in Fig. 2(b) reflects the *R*_ct_ and series resistance (*R*_s_) whose exact values are obtained by fitting the equivalent circuit in the inset of Fig. 2(b). As shown in Table 1, the *R*_ct_ values were reduced after substituting Zn^2+^ by Co^2+^ and Ni^2+^ ions (CCTS, 5.3 Ω; CNTS, 5.5 Ω; CZTS, 6.5 Ω), which was consistent with the variation of *J*_0_ values in Fig. 2(a). In addition, the *R*_s_ values of CCTS and CNTS CEs were close to that of CZTS, indicated their similar electron transport ability. Thus, the variation of *R*_ct_ led to the enhancement of the catalytic activity of CCTS and CNTS CEs, compared with CZTS. This catalytic activity trend was also observed on dense CXTS films prepared by spin-coating the dimethyl sulphoxide-based precursor solution (see Fig. S3 in the Supplementary Information). All the electrochemical data suggested that the substitution of Zn^2+^ by Co^2+^ and Ni^2+^ effectively improved the electrocatalytic activity of kesterite CZTS CEs for reducing triiodides.

**Table 1:** Electrochemical parameters of EIS plots with Pt and CXTS CEs and photovoltaic parameters obtained from DSSCs with Pt and CXTS CEs.

CE	*R*_s_ (Ω)	*R*_ct_ (Ω)	*J*_sc_ (mA cm^−2^)	*V*_oc_ (V)	FF	PCE (%)
Pt	12.4	5.9	16.80	0.68	0.73	8.3
CZTS	13.1	6.5	16.05	0.67	0.73	7.9
CCTS	12.9	5.3	16.79	0.68	0.72	8.3
CNTS	12.9	5.5	16.74	0.67	0.73	8.2

### Photovoltaic performance of DSSCs

The current density-voltage (*J*–*V*) curves of DSSCs containing Pt or CXTS CEs and N719-sensitized TiO_2_ photoanode in iodide electrolyte were shown in Fig. 2(c). Table 1 summarizes the resultant photovoltaic parameters. The CCTS- and CNTS-based DSSCs revealed comparable power conversion efficiency (PCE) (8.3% and 8.2%, respectively) with that of Pt-based DSSC (8.3%), which were derived from the short-circuit current density (*J*_sc_) of 16.79 and 16.74 mA cm^−2^, open-circuit voltage (*V*_oc_) of 0.68 V and 0.67 V, and fill factor (FF) of 0.72 and 0.73. The CCTS and CNTS cells exhibit a higher *J*_sc_ and PCE than that of CZTS (*J*_sc_, 16.05 mA cm^−2^; PCE, 7.9%) owing to the higher activity of the CCTS and CNTS CEs. In addition, photovoltaic parameters of five parallel CXTS-based DSSCs indicate the good repeatability of CXTS CEs (see Fig. S4 and Table S1 in the Supplementary Information). Therefore, the electrochemical data of dummy cells and photovoltaic performance of DSSCs confirm that substitution of Zn^2+^ by Co^2+^ and Ni^2+^ is effective for improving the electrocatalytic ability of kesterite CZTS CEs.

### Density functional theory calculation

**Figure 3:**
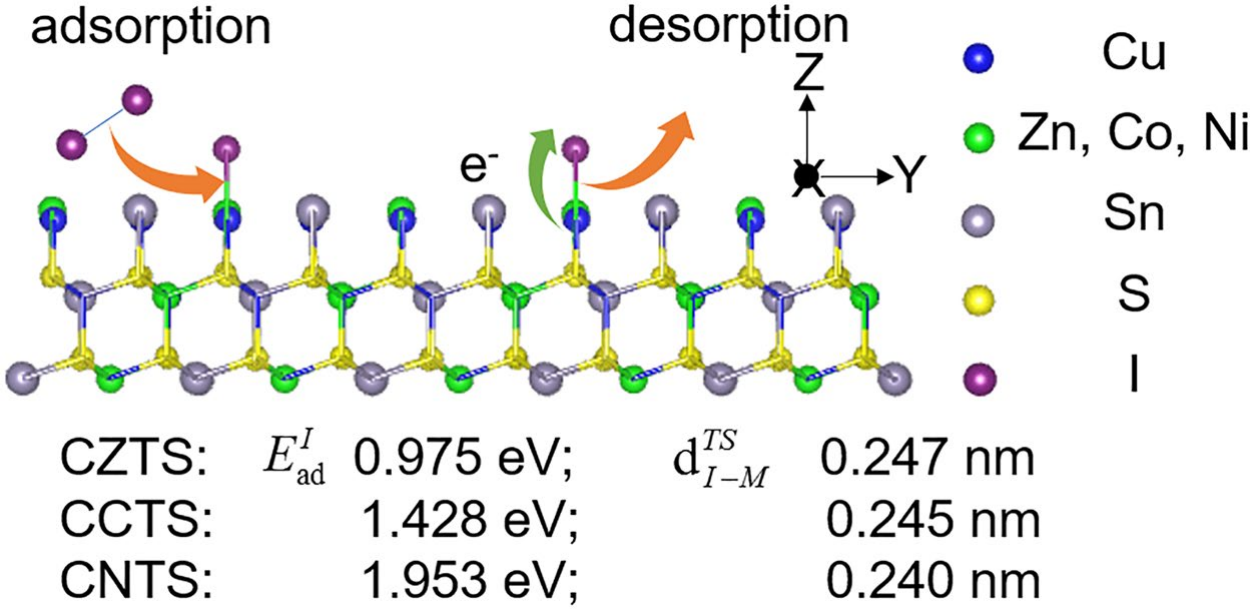
The schematic diagram of reducing triiodides on CXTS (112) surface: adsorption of I_2_, electron transfer, desorption of I^*^. The corresponding adsorption energy ${{\rm{E}}}_{{\rm{ad}}}^{I}$, bond length ${{\rm{d}}}_{I-M}^{{\rm{TS}}}$ are also shown.

Considering that the catalytic activity of CEs strongly correlates with the adsorption and desorption processes of redox species, we perform density functional theory calculation to explore the origin of catalysis-activity enhancement caused by element substitution. First, we checked the change of adsorption energy toward I atom (${{\rm{E}}}_{{\rm{ad}}}^{I}$) during the substitution of Zn^2+^ by Co^2+^ and Ni^2+^. We found that the I atom was preferentially adsorbed on Zn^2+^ of CZTS, as the calculated ${{\rm{E}}}_{{\rm{ad}}}^{I}$ value of Sn^4+^ (0.295 eV) was significantly lower than that of Zn^2+^ (0.975 eV) (see Fig. S5 in the Supplementary Information). And the ${{\rm{E}}}_{{\rm{ad}}}^{I}$ value (Fig. 3 and Fig. S5 in the Supplementary Information) remarkably increased to 1.428 eV (CCTS) and 1.953 eV (CNTS) after the element substitution. This change indicated the stronger adsorption ability toward I atom of CCTS and CNTS CEs, resulted in their more efficient catalytic activity for reducing triiodides. Moreover, the calculated bond length between I atom and metal ions for the transition state (${{\rm{d}}}_{I-M}^{{\rm{TS}}}$) decreased from 0.247 nm of CZTS to 0.245 nm of CCTS and 0.240 nm of CNTS, which could result in more difficult desorption of the adsorbed I atom (I^*^). These theoretical calculation data showed that the enhanced performance of CCTS and CNTS CEs compared CZTS was associated to the improved adsorption and desorption energy.

**Figure 4:**
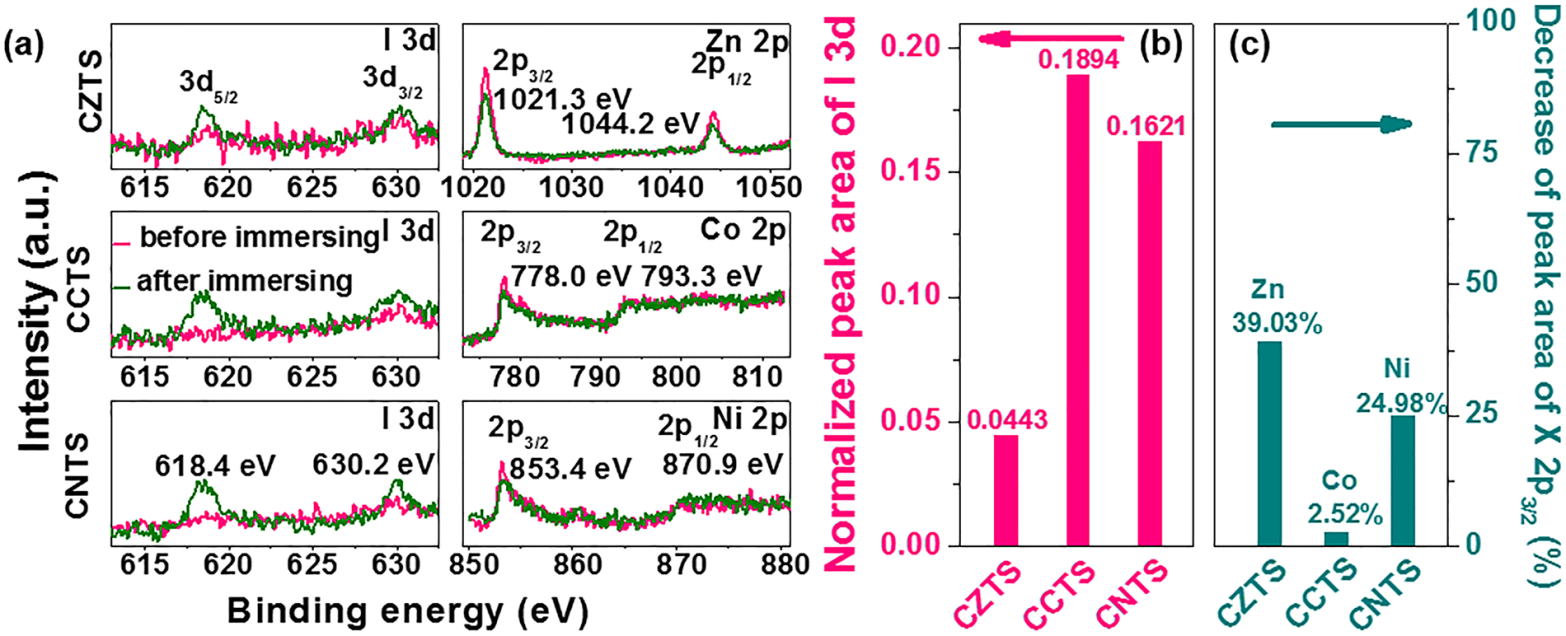
(**a**) I 3d, Zn 2p, Co 2p and Ni 2p XPS spectra of CXTS CEs before and after immersing in the iodide electrolyte for 30 minutes. (**b**) The normalized peak area of I 3d XPS spectra of CXTS CEs after immersing in the iodide electrolyte for 30 minutes and (**c**) the decrease of peak area of X 2p_3/2_ XPS spectra of CXTS CEs after immersing.

Furthermore, we compared the amounts of I atom adsorbed on the CXTS surface by XPS^57–60^ (see Fig. S6 in the Supplementary Information and Fig. 4). We immersed CXTS CEs in the iodide electrolyte for 30 minutes and rinsed them with ethanol. The peak area ratio of I 3d to Cu 2p spectra was marked as the normalized peak area of I 3d spectra. No signals of I 3d were found in XPS results before immersing. But, after immersing, the normalized peak area of I 3d spectra of CCTS (0.1894) and CNTS (0.1621) were significantly larger than that of CZTS (0.0443) (Fig. 4(b)), indicating more I atom adsorbed on CCTS and CNTS CEs surface. This change was consistent with the enhanced ${{\rm{E}}}_{ad}^{{\rm{I}}}$ values and decreased bond length. The electrochemical, photovoltaic and theoretical results all indicated that the substitution of Zn^2+^ by Co^2+^ and Ni^2+^ was effective to improve the catalytic activity of kesterite CZTS.

### Durability test

**Figure 5:**
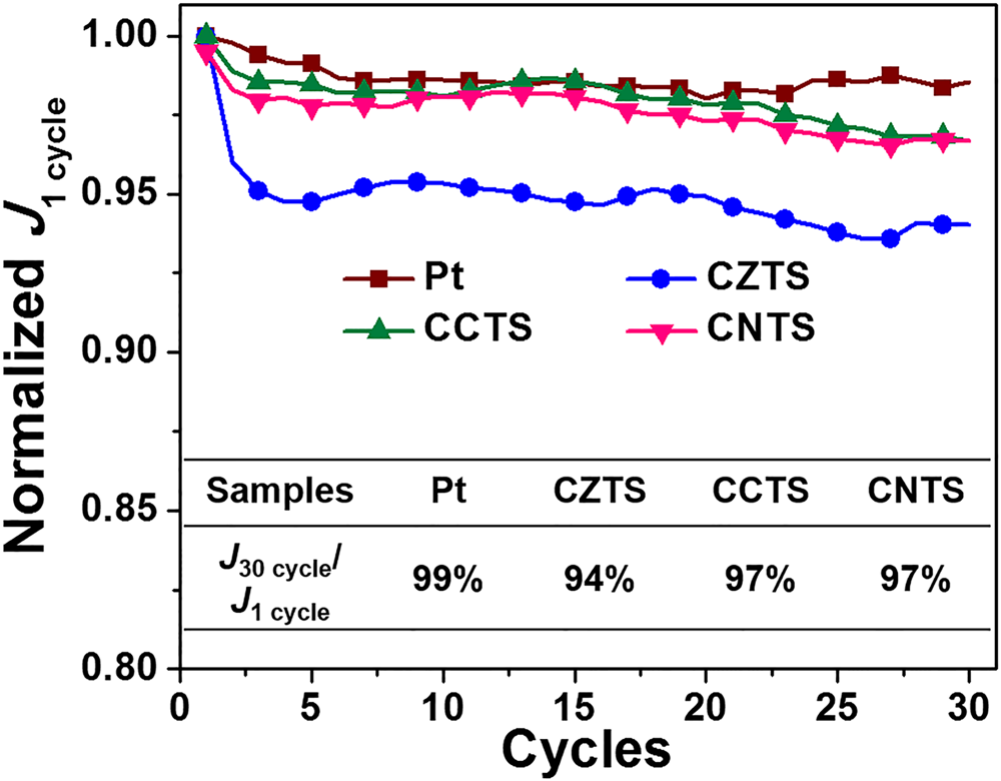
The change of the current density under −0.40 V for different cycles of Tafel polarization measurement of Pt and CXTS CEs. The current density of the first cycle and the last cycle are marked as *J*_1_ cycle and *J*_30_ cycle, respectively.

The stability is one of the major factors to evaluate the property of CEs^61–63^. Herein, we used Tafel, EIS and XPS tests to examine the stability of CXTS CEs. First, the current density in Tafel curves at −0.40 V (see Fig. S7 in the Supplementary Information) of the CZTS CEs decreased by 6% compared with the original ones after 1800 s test (Fig. 5). Whereas, the current density of Pt, CCTS and CNTS CEs only decreased by less than 3%, indicating the better stability of CCTS and CNTS CEs. The fitted *R*_s_ and *R*_ct_ values obtained from EIS spectra of CXTS CEs before and after immersing in the iodide electrolyte for 30 minutes (see Fig. S8 and Table S2 in the Supplementary Information) also showed the good stability of CCTS and CNTS. In addition, after immersing, the peak area of Co and Ni XPS spectra decreased by 2.52% and 24.98% of the original ones, respectively (Fig. 4(c)). This result was significantly smaller than that of Zn spectra in CZTS (39.03%). Different stability measurements all suggested that the CCTS and CNTS CEs possessed better stability toward the iodide electrolyte compared with CZTS CE.

## Conclusions

In conclusion, we proved that the substitution of Zn^2+^ by Co^2+^ and Ni^2+^ was a convenient but effective approach to enhance the electrocatalytic performance of kesterite CZTS CEs in DSSCs. After substitution, CCTS and CNTS CEs exhibited decreased charge transfer resistance (CCTS, 5.3 Ω; CNTS, 5.5 Ω; CZTS, 6.5 Ω) and improved electrocatalytic activity (PCE: CCTS, 8.3%; CNTS, 8.2%) compared with CZTS (7.9%) toward iodide electrolyte, which was comparable with the traditional Pt-based cells (8.3%). The enhanced activity was associated to the change of adsorption and desorption energy (the bond length between I atom and metal ions for the transition state (${{\rm{d}}}_{I-M}^{{\rm{TS}}}$) of I atom by theoretical calculation. Furthermore, the stability of kesterite CXTS CEs was also significantly improved. The results indicated that this element substitution method without changing the materials structure was effective to improve potential catalysts performance, especially for the multicomponent compounds.

## Electronic supplementary material

Supplementary Materials
