# Supplementary material for: Element substitution of kesterite Cu2ZnSnS4 for efficient counter electrode of dye-sensitized solar cells
Source: Sci Rep. 2018 Jun 7;8:8714. doi: 10.1038/s41598-018-26770-1 (PMC5992223; doi:10.1038/s41598-018-26770-1)
Supplement: Supplementary file 1 — Supplementary Materials [file 41598_2018_26770_MOESM1_ESM.pdf]

Supporting Information

Element substitution of kesterite Cu_2ZnSnS_4 for efficient counter electrode of dye-sensitized solar cells

Shuang Lu, Huanying Yang, Fei Li, Yinglin Wang*, Shixin Chen, Guochun Yang, Yichun Liu & Xintong Zhang*

Center for Advanced Optoelectronic Functional Materials Research, and Key Lab of UV-Emitting Materials and Technology of Ministry of Education, Northeast Normal University, 5268 Renmin Street, Changchun 130024, China

*Corresponding authors:

Yinglin Wang: wangyl100@nenu.edu.cn.

Xintong Zhang: xtzhang@nenu.edu.cn.

Preparation of CXTS (CZTS, CCTS, and CNTS) films

Referring to the previously published method, we made several changes to prepare CXTS films, CXTS precursor solution was first prepared by dissolving $CuCl_2{\cdot}2H_2O$ (0.16 M), $ZnCl_2$ ($CoCl_2{\cdot}6H_2O$ or $NiCl_2{\cdot}6H_2O$) (0.12 M), $SnCl_2{\cdot}2H_2O$ (0.1 M) and thiourea (0.67 M) in a mixture solution containing water and ethanol (1:2, v/v), then 0.125 ml 3-mercaptopropionic acid was added into the solution. After stirring about 5 min, a clear CXTS precursor solution was obtained. CXTS films were obtained on well-cleaned SnO_2: F (FTO) glass (2.25 cm^2) by spin-coating the precursor solution at 2500 rpm for 30 s, followed by drying at 300 °C for 1 min. Then, the prepared CXTS films were annealed at 540 °C for 15 min in N_2 atmosphere.

Fabrication of DSSCs

In a typical procedure, mesoporous TiO_2 layer (active area: 0.126 cm^2) was prepared by a screen-printing method, after being sintered at 500 °C for 30 min and cooled to room temperature, the TiO_2 electrode was immersed in a dry ethanol solution containing 0.3 mM of N719 at room temperature for 24 h to complete dye adsorption. The prepared CXTS and traditional Pt films prepared by thermal deposition of H_2PtCl_6 acted as counter electrodes. An acetonitrile/valeronitrile (85:15, v/v) electrolyte (I^-/I_3^-) containing 0.6 M 1,3-dimethylimidazolium iodide, 0.1 M guanidinium thiocyanate, 0.03 M iodine, 50 mM lithium iodide, and 0.5 M 4-tert-butylpyridine was injected into the space between the TiO_2 and counter electrodes.

Characterizations

X-ray diffraction patterns were recorded on a Rigaku, D/max-2500 X-ray diffractometer. Raman spectra were obtained by J-Y UV-lamb micro-Raman spectrometer under an excitation of a 488 nm Ar^+ laser. Scanning electron microscope images and energy-dispersive X-ray (EDX) spectra of the samples were observed via an FEI Quanta 250 field-emission. UV-vis spectra were recorded

with a Hitachi UH 4150 UV-visible-NIR spectrophotometer. The atomic force microscopy (AFM) images were recorded via a Dimension Icon atomic force microscope produced by Bruker. X-ray photoelectron spectroscopy experiments were performed on a VGESCA-LAB MKII instrument with an Al Kα ADES (hν=1486.6 eV) source. Photocurrent density-voltage (*J*–*V*) characteristics of the solar cell were measured under AM 1.5G simulated solar light (ABET Technology, 100 mW cm^{-2}) standard by a standard silicon reference cell. For Tafel polarization curves and electrochemical impedance spectroscopy, symmetrical cells consisting of two identical electrodes were measured by using a Princeton PARSTAT 2273 potentiostat/galvanostst. Tafel polarization curves were conducted at 50 mV s^{-1} under the bias between −1 and 1 V. Electrochemical impedance spectroscopy was obtained under a bias of 0.4 V, the frequency ranged from 100 mHz to 600 kHz.

Computational Methods

Structural relaxations and total energy calculations were performed in the framework of density functional theory within the generalized gradient approximation (GGA) as implemented in the Vienna ab initio simulation program package (VASP)[1-3]. Electron-ion interactions were described using standard PAW potentials[4,5], with valence configurations of $3d^84s^1$ for Co, $3d^84s^2$ for Ni, $3d^{10}4p^1$ for Cu, $3d^{10}4p^2$ for Zn, $5s^25p^2$ for Sn and $5s^25p^5$ for I. A cutoff energy of 400 eV was used. Herein Perdew-Burke-Ernzerhof (PBE)[6] functional were used for the GGA-level DFT calculations. We used a periodic cell containing 104 atoms of Cu_2XSnS_4 (X = Co, Ni and Zn). Due to the insufficient consideration of the on-site Columbic repulsion, between the d electrons of Co, Ni and Cu, DFT may fail to describe the electronic structure of Cu_2XSnS_4. To overcome this shortcoming,

the GGA+U approach was adopted[7]. U–J = 6.0 eV for Co, Ni and Cu atoms was used. To accurately simulate the Cu_2XSnS_4 (112) surface, a 7-layer slab was enclosed with a sufficiently large vacuum region of 15 Å to ensure the periodic images to be well separated. During the structural relaxations, the atoms in the bottom five layers were fixed and local optimizations were stopped when the enthalpy changes became smaller than $1x10^{-4}$ eV per cell. Due to the inherent magnetism of Cu_2XSnS_4, spin-polarization calculation was performed. For Brillouin-zone integrations, gamma-centered k-point grids of special points with a (3 x 2 x 1) mesh were used for the calculation of I adsorption on Cu_2XSnS_4 (112) surface and the surface cell[8].

Data Availability. The datasets generated during and/or analyzed during the current study are available from the corresponding author on reasonable request.

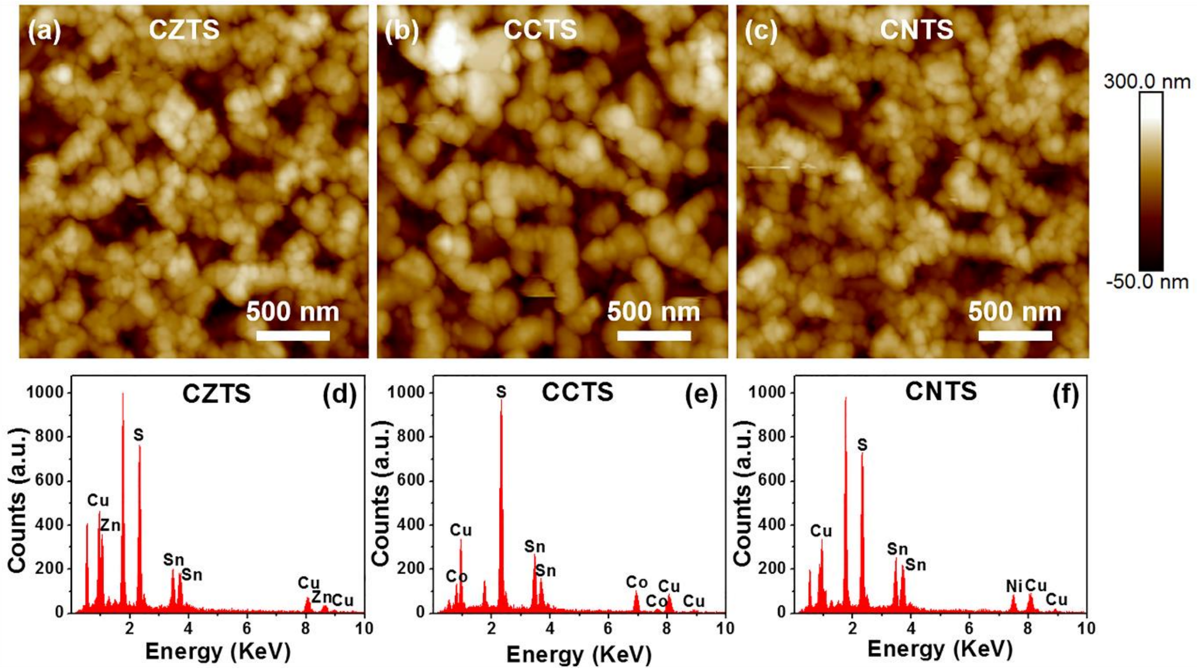

Figure S1. (a, b and c) The EDX spectra and (d, e and f) AFM images of CXTS films.

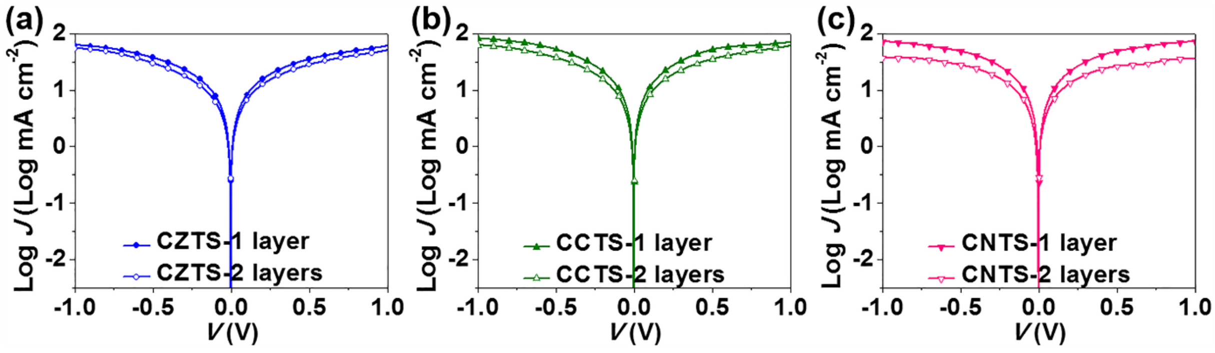

Figure S2. (a, b and c) Tafel polarization curves of CXTS CEs with 1 layer and 2 layers.

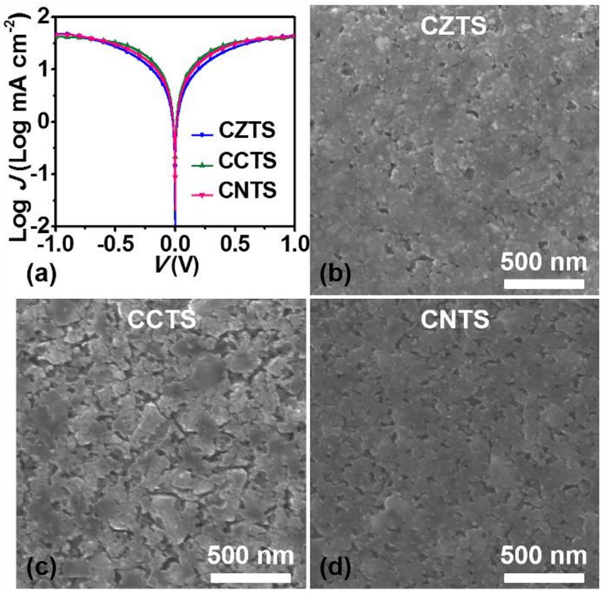

Figure S3. Tafel curves and top-view SEM images of CXTS films prepared by dimethyl sulphoxide precursor solutions.

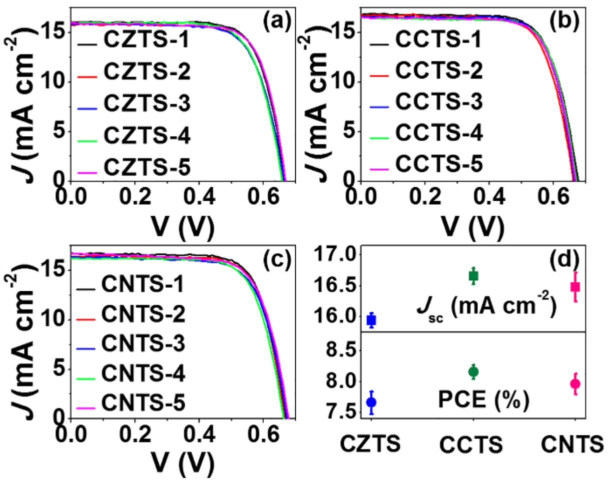

Figure S4. (a, b and c) The *J–V* curves of five parallel DSSCs based on CXTS CEs. (d) Average and standard deviation of J_{sc} and PCE obtained from five parallel CXTS-based DSSCs.

Table S1.

The average photovoltaic parameters and standard deviation of five parallel CXTS-based DSSCs in Figure S4.

Cells	J_{sc} (mA cm^{-2})	V_{oc} (V)	FF	PCE (%)
CZTS	15.94±0.11	0.67±0.01	0.72±0.11	7.66±0.18
CCTS	16.65±0.13	0.67±0.01	0.72±0.01	8.16±0.11
CNTS	16.48±0.26	0.67±0.01	0.72±0.05	7.96±0.17

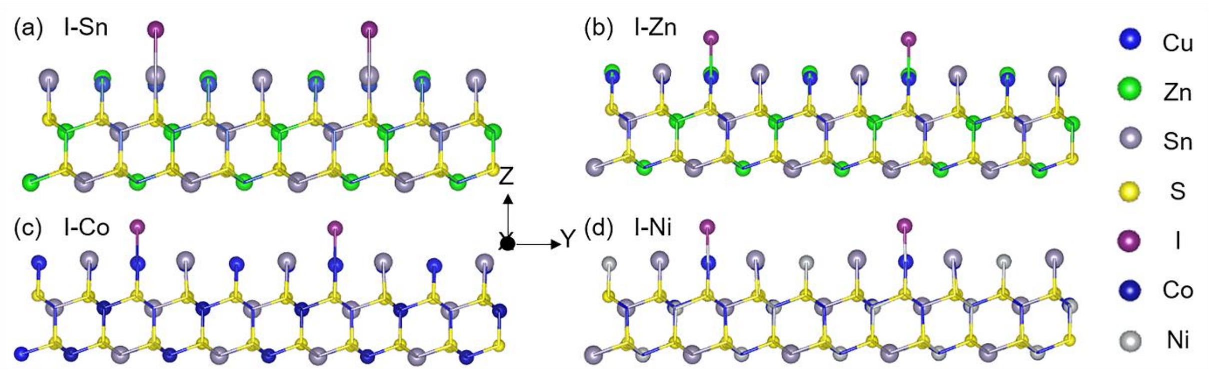

Figure S5. (a, b, c, and d) Atom arrangements diagrams of iodine atom adsorbed on Sn, Zn, Co, and Ni atom of CXTS (112) surface.

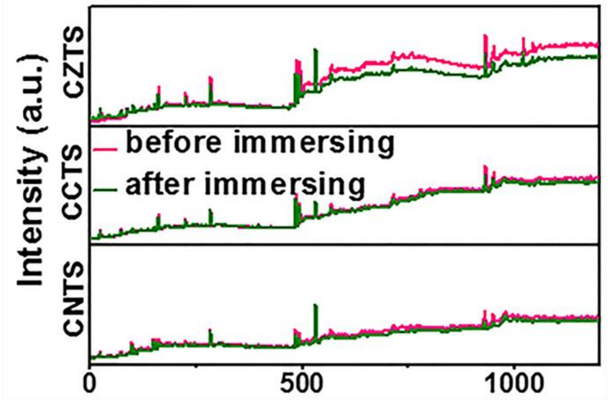

Figure S6. Survey XPS spectra of CXTS CEs before and after immersing in the iodide electrolyte for 30 minutes. The binding energy is corrected referencing C 1s (284.60 eV).

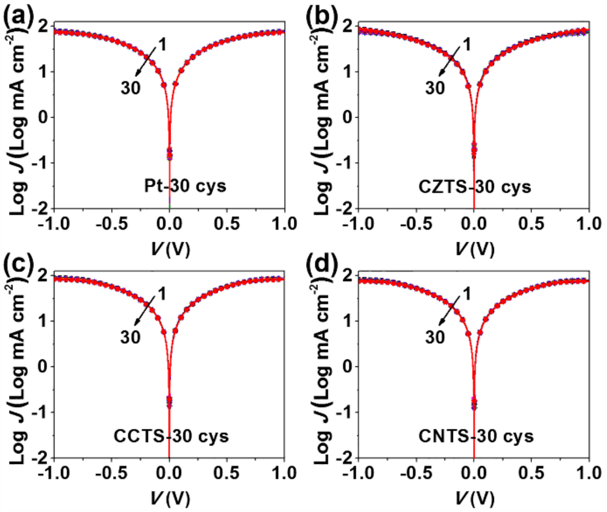

Figure S7. Tafel curves of Pt and CXTS CEs in symmetrical dummy cells for continuous test.

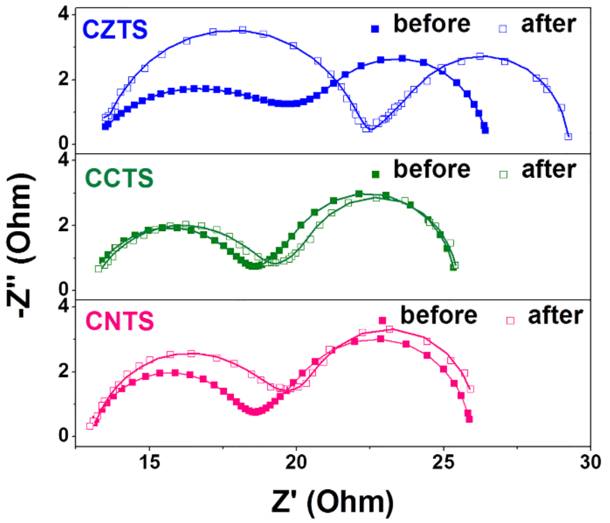

Figure S8. EIS plots of CXTS CEs before and after immersing in the iodide electrolyte for 30 minutes.

Table S2.

The fitted R_s and R_{ct} obtained from EIS plots of CXTS CEs after immersing in the iodide electrolyte for 30 minutes.

Cells	R_s (Ω)	R_{ct} (Ω)
CZTS	13.3	9.0
CCTS	13.0	6.1
CNTS	13.1	6.3

References

1. Kresse, G. & Hafner, J. Ab. initio molecular dynamics for liquid metals, *Phys. Rev. B: Condens. Matter Mater. Phys.* **47,** 558-561 (1993).

2. Kresse, G. & Furthmüller, J. Efficiency of ab-initio total energy calculations for metals and semiconductors using a plane-wave basis set, *Comput. Mater. Sci.* **6,** 15-50 (1996).

3. Kresse, G. & Joubert, D. From ultrasoft pseudopotentials to the projector augmented-wave method, *Phys. Rev. B: Condens. Matter Mater. Phys.* **59,** 1758-1775 (1999).

4. Vanderbilt, D. Soft self-consistent pseudopotentials in a generalized eigenvalue formalism, *Phys. Rev. B: Condens. Matter Mater. Phys.* **41,** 7892-7895 (1990).

5. Kresse, G. & Hafner, J. Norm-conserving and ultrasoft pseudopotentials for first-row and transition elements, *J. Phys.: Condens. Matter* **6,** 8245-8257 (1994).

6. Perdew, J., Burke, K. & Ernzerhof, M. Generalized Gradient Approximation Made Simple, *Phys. Rev. Lett.* **77,** 3865-3868 (1996).

7. Dudarev, S., Botton, G., Savrasov, S., Humphreys, C. & Sutton, A. Electron-energy-loss spectra and the structural stability of nickel oxide: An LSDA1U study, *Phys. Rev. B: Condens. Matter Mater. Phys.* **57,** 1505-1509 (1998).

8. Yu, X. *et al.* Cu_2ZnSnS_4 Nanocrystals as Highly Active and Stable Electrocatalysts for the Oxygen Reduction Reaction, *J. Phys. Chem. C* **120,** 24265-24270 (2016).
